# *nagZ* Triggers Gonococcal Biofilm Disassembly

**DOI:** 10.1038/srep22372

**Published:** 2016-03-01

**Authors:** Senthil V. Bhoopalan, Andrzej Piekarowicz, Jonathan D. Lenz, Joseph P. Dillard, Daniel C. Stein

**Affiliations:** 1 University of Maryland Department of Cell Biology and Molecular Genetics, College Park, MD, 20742, USA; 2 University of Wisconsin-Madison School of Medicine and Public Health, Department of Medical Microbiology and Immunology, Madison, WI, 53792, USA

## Abstract

Bacterial-bacterial interactions play a critical role in promoting biofilm formation. Here we show that NagZ, a protein associated with peptidoglycan recycling, has moonlighting activity that allows it to modulate biofilm accumulation by *Neisseria gonorrhoeae*. We characterize the biochemical properties of NagZ and demonstrate its ability to function as a dispersing agent for biofilms formed on abiotic surfaces. We extend these observations to cell culture and tissue explant models and show that in *nagZ* mutants, the biofilms formed in cell culture and on human tissues contain significantly more biomass than those formed by a wild-type strain. Our results demonstrate that an enzyme thought to be restricted to peptidoglycan recycling is able to disperse preformed biofilms.

Gonorrhea, caused by the exclusive human pathogen *Neisseria gonorrhoeae* (GC), is the second most common reportable bacterial disease in the US, affecting over 800,000 people every year^1^. While laboratory studies have contributed to a significant understanding of the mechanistic underpinnings of GC interactions with the host, during natural infection the organism probably exists on mucosal surfaces as a biofilm^2^. This observation leads to questions about potential differences in studies done with planktonic bacteria as compared to how the organism is growing *in vivo*. GC growth in biofilms also has implications for treatment, especially with the emergence of cefixime and ceftriaxone-resistant strains^3^,^4^.

GC can form a biofilm on glass, primary and transformed human cervical epithelial cells^2^. However, little is known about how gonococci form biofilms, the signals that regulate biofilm production or the mechanism by which gonococci disperse from the biofilm. DNA plays a critical role in organizing the neisserial biofilm matrix, with secretion of single-stranded DNA playing an important role in the initiation of gonococcal biofilms^5^. However, the source of this DNA is unclear as strains such as FA1090 and 1291 form biofilms, yet lack the type 4 secretion system associated with DNA secretion^6^. Addition of exogenous DNase or Spermine impairs biofilm formation and contributes to the disruption of the biofilm^7^,^8^. Since the human urogenital track is rich in polyamines, and these molecules can protect GC from host antimicrobials^9^, interactions with polyamines may be one mechanism by which GC leave a biofilm under physiological conditions. Studies on biofilms produced by other organisms have suggested several mechanisms of dispersal, including erosion^10^ and sloughing^11^. Erosion and sloughing can result from bacterial metabolism-associated processes, such as the secretion of glycosidases^12^ and nucleases^13^.

GC in biofilms appear to be intimately associated with each other through unknown mechanisms. Our previous studies demonstrated that *N. gonorrhoeae* MS11ΔOpa, a strain that lacks opacity protein expression, is impaired in its ability to form microcolonies^14^. Since Opa proteins can act as lectins and bind to LOS from neighboring cells^15^, we hypothesized that there must be a glycosidase that modulates LOS-Opa interactions to allow for separation individual cocci from a microcolony. We further predicted that this protein could regulate bacterial escape from a biofilm.

In this study, we characterized NagZ from *N. gonorrhoeae* and analyzed its role in biofilm development. We expressed *nagZ* in *Escherichia coli,* purified the protein and defined its biochemical properties. We show that a *nagZ* mutant produces a more robust biofilm that accumulates more biomass over time. We also show that exogenously added NagZ reduces the biofilm. Finally, we show that in the absence of NagZ, gonococci produce thicker biofilms on *ex vivo*-cultured human cervical tissue.

## Results

### NagZ is a non-essential β-*N*-acetylglucosaminidase

Using the CAZy database^16^, we determined that ORF0135 in *N. gonorrhoeae* FA1090 encoded a putative beta-hexosaminidase (EC 3.2.1.52) belonging to glycoside phosphorylase (GH3) superfamily. Glycosidases from GH3 are retaining enzymes and cleave their substrates in an acid/base-catalyzed two-step double-displacement mechanism involving a covalent glycosyl-enzyme intermediate in which a fully conserved aspartic acid functions as the catalytic nucleophile^17^. Since ORF0135 is homologous to NagZ in a wide variety of gram-negative bacteria and contains the conserved aspartic acid (the homology to *E. coli* and *Pseudomonas aeruginosa* is shown in Supplemental Fig. 1) we propose renaming ORF0135 *nagZ.* This gene is present in all neisserial genomes currently available in NCBI, with a high degree of conservation (>97% identity) at the nucleotide level among GC strains (Figure S1). The gene synteny is identical in the pathogenic strains, but diverges significantly in commensal strains (data not shown).

The DNA encoding NagZ was cloned into the expression vector pET28a and the expressed protein purified. The molecular mass of NagZ as determined by SDS-PAGE (47 kDa) was consistent with the predicted mass for this protein (Supplemental Fig. 2). We determined the optimal enzymatic conditions for NagZ using *p*-Nitrophenyl *N*-acetyl-β-D-glucosaminide (pNP-GlcNAc) as a substrate. The optimal conditions for the assay were determined to be 37 °C, in KPO_4_ buffer (200 to 400 mM) at a pH of 8.0 (Supplemental Fig. 2B,C). The enzyme retained 100% of activity after 48 hr at room temperature (data not shown). The kinetic parameters *K*_m_ and V_max_ against (pNP-GlcNAc) were 3.2 mM and 64 μmol min^−1^ mg^−1^ respectively, and these values are in the same order of magnitude as those for several other hexosaminidases^18^. We calculated the specific activity of our purified protein as 1600 nmol min^−1^ mg^−1^. We screened for activity of this enzyme using *p-*Nitrophenyl conjugated to other sugar moieties (Supplemental Fig. 2D). Of the substrates that produced detectable cleavage products, NagZ had about 20% maximal activity on *p*-nitrophenyl-beta-D-*N,N*’-diacetylchitobiose versus that measured with PNP-GlcNAc.

Because *N. gonorrhoeae* lacks the genes needed to encode a carbohydrate capsule^19^, cell surface *N*-acetyl-glucosamine is only found associated with LOS. However, this would not preclude NagZ possessing the ability to cleave *N*-acetyl-glucosamine linked through other conformations. This led us to test *Staphylococcus aureus,* which produces an extracellular polysaccharide that is a β-1-6-linked polymer, predominately composed of *N*-acetyl-glucosamine^20^ that is required for biofilm formation^21^. When this polymer is degraded by treatment with dispersin B, the biofilm is disrupted^22^. We tested to see if NagZ could disrupt a biofilm formed by *S. aureus* SH1000. NagZ treatment of a *S. aureus* biofilm had a negligible impact on its biofilm (Supplemental Fig. 2E). Conversely, treatment with Dispersin B significantly reduced the biomass of the *S. aureus* biofilm. Dispersin B treatment of gonococcal biofilms produced a negligible impact on its biofilm (data not shown). Given that prolonged treatment of a *S. aureus* biofilm with NagZ failed to have an impact on it, it is likely that NagZ lacks exo- and endo-β-1-6- N-acetyl-glucosaminidase activity.

Because NagZ is needed for the formation of monosaccharides from the released disaccharides during the cytosolic steps of the muropeptide-recycling pathway in *E. coli*^23^, we hypothesized that the accumulation of peptidoglycan fragments that would occur in a *nagZ* mutant could affect gonococcal viability. We used a recombination strategy to delete this gene and verified that the deletion had been incorporated properly in the genome by DNA sequence analysis of the region containing the deletion. We measured the growth properties of the deletion mutant and the data indicate that in standard liquid growth media, there is no difference in growth between the parent and deletion strain (Supplemental Fig. 3A).

NagZ activity could be detected in the supernatant as the culture enters stationary phase (Supplemental Fig. 3A). We could not detect any NagZ activity in culture supernatants of Log-phase cells, but could readily detect its activity in the cell lysate. Bioinformatic programs to predict the cellular location of *nagZ* (Signal p4.0^24^, PSORTb v3.0^25^, PSLPred^26^ and MESSA^27^) suggested a cytosolic or periplasmic location for NagZ (data not shown). Using both bacterial lysates and broth supernatants isolated from overnight cultures from FA1090∆nagZ, we were unable to detect beta-hexosaminidase activity, suggesting that NagZ is the only beta-hexosaminidase in *N. gonorrhoeae* FA1090.

### NagZ acts on peptidoglycan

**Figure 1:**
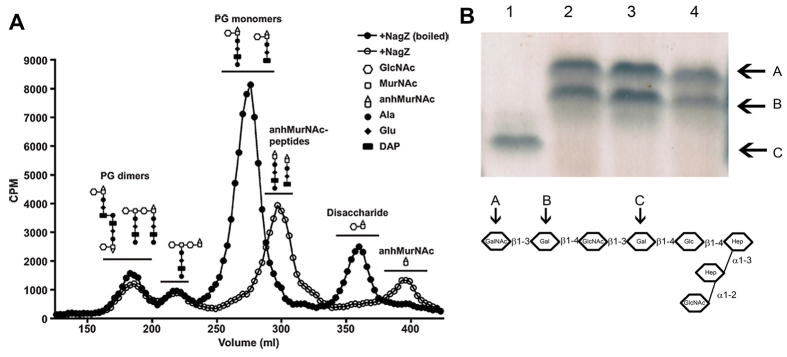
Enzymatic specificity of NagZ on neisserial components. Panel (**A**) demonstrates NagZ activity on peptidoglycan. Gonococci were metabolically labeled with [6-^3^H]glucosamine. After a chase period, supernatants were collected and treated with purified NagZ. Radiolabeled fragments were separated by size exclusion chromatography and detected by liquid scintillation counting. Gonococcal peptidoglycan contains PG dimers, monomers, free disaccharide, and anhydro-*N*-acetylmuramic acid. GlcNAc, *N*-acetylglucosamine; MurNAc, *N*-acetylmuramic acid; anhMurNAc, anhydro-*N*-acetylmuramic acid. Panel (**B**) is an SDS-PAGE gel of LOS isolated from: 1) *N. gonorrhoeae* F62 Δ8-1^42^; 2) *N. gonorrhoeae* F62; 3) *N. gonorrhoeae* F62ΔNagZ; and 4) *N. gonorrhoeae* F62ΔNagZ treated with NagZ. The lower portion of the panel is a graphical representation of the oligosaccharide portion of LOS isolated from F62^51^. Letters above the arrows correspond to the LOS bands seen in the upper panel.

As gonococci grow they recycle the peptidoglycan fragments removed from the sacculus. However, they release a significant portion of these fragments into the medium, and characterization of these fragments produces a reproducible chromatographic profile^28^. We tested NagZ for its ability to digest metabolically-labeled peptidoglycan fragments. Using a boiled preparation of NagZ as a negative control, we generated the expected profile (Fig. 1). The data in Fig. 1A show that the addition of NagZ altered the profile of peptidoglycan fragments, where the peptidoglycan monomer peak shifted to later fractions (smaller size) from the sizing columns, consistent with the removal of N-acetylglucosamine from the peptidoglycan monomers. Similarly, the free disaccharide peak was eliminated and a peak appeared for free 1,6-anhydro-N-acetylmuramic acid. No significant changes were seen to the peaks for peptidoglycan dimers and tetrasaccharide-peptide, suggesting that these molecules are not substrates of NagZ. These data are consistent with the known activity of NagZ in other bacteria, acting to remove N-acetylglucosamine from peptidoglycan monomers and free disaccharide^29^.

### NagZ does not act upon LOS

Because NagZ was able to cleave substrates containing *N*-acetyl-glucosamine, and neisserial LOS contains *N*-acetylglucosamine in a variety of conformations, we assayed NagZ for its ability to act upon neisserial LOS. Purified LOS from *N. gonorrhoeae* F62 was digested with NagZ and its mobility compared with undigested LOS. Because F62 expresses several different isoforms of LOS containing terminal and internal N-acetylglucosamine, and NagZ was unable to act on any of these components, whether at a terminal or internal location (Fig. 1B), we concluded that NagZ lacks β1–3 exoglycosidase activity and α1–2 endoglycosidase activity. We also performed these assays by adding NagZ to intact cells, and analyzing the LOS after treatment. The data obtained was the same as that obtained using purified LOS, indicating that the enzymatic activity is not linked to the presence of a specific LOS conformation (Data not shown).

### The *nagZ* deletion strain forms a thicker biofilm

**Figure 2:**
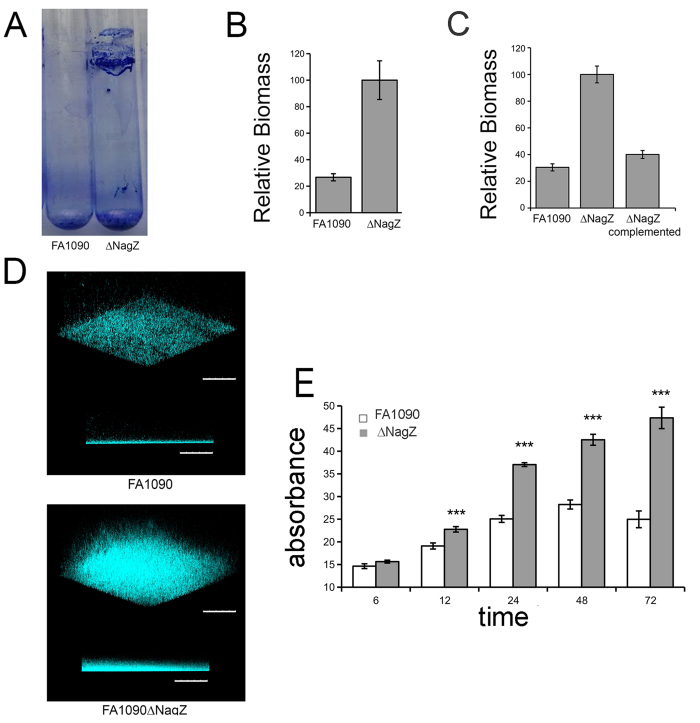
Biofilm formation by gonococcal strains. Panel (**A**) A photograph of dynamic biofilm formed by wildtype bacteria (left) and the mutant strain (right) after 24 hr. Panel (**B**) Quantification of dynamic biofilm formation (n = 6). Panel (**C**) Complementation of *nag*Z in the knock out strain reduced the ability of the mutant to form a thicker biofilm in a static biofilm (n = 5). Panel (**D**) Confocal imaging of 24 hour old static biofilm, after staining with Hoescht stain. The mutant strain forms a thicker and denser biofilm. Scale bar represents 100 μm. Panel E: Static biofilms were prepared, and the biomass of the biofilm determined at various time points. Data represents mean values (±SE) of three independent experiments performed in triplicate. Statistics were two tailed t-test (***p < 0.001). ns = Not significant.

Gonococcal strains growing in biofilms are under significant metabolic stress^30^. As such, we would expect that the extracellular milieu of a biofilm would contain cytosolic enzymes because stressed cells are dying and dying cells are lysing. We hypothesized that a *nagZ* mutation could alter the structure of a gonococcal biofilm. The wild type and Δ*nagZ* strains of *N. gonorrhoeae* FA1090 were compared for their ability to form a biofilm under static and dynamic conditions. Dynamic biofilms were developed at the liquid air interface on the inner surface of culture tubes. After 24 hr, the bacterial culture was aspirated and the biofilm dried. The data (Fig. 2, panel A) is a visual representation of the biofilm that was formed on a culture tube. The data in Fig. 2, panel B is a quantification of the biomass of this biofilm, using a crystal violet staining procedure^31^. Statistical analysis using Student’s t-test showed a significant increase in biomass of the knockout strain over the wild type strain (n = 6, p < 0.001) of approximately 4 fold. A similar phenotype was observed when biofilms were made under static conditions (Fig. 2C). This biofilm enhancement was lost when the mutant was complemented (Fig. 2C). We performed confocal microscopy on static biofilms grown for 36 hr, using Hoechst stain to visualize the biofilm. The mutant strain shows a denser and taller biofilm (~4 fold), consistent with quantification of biomass using crystal violet (Fig. 2D). We compared the ability of FA1090 and FA1090∆nagZ to form biofilms over time. The biomass associated with FA1090∆nagZ continued to accumulate over 3 days, while the biomass of a biofilm produced by FA1090 peaked around 48hr (Fig. 2E).

### Effect of NagZ on gonococcal biofilm formation

**Figure 3:**
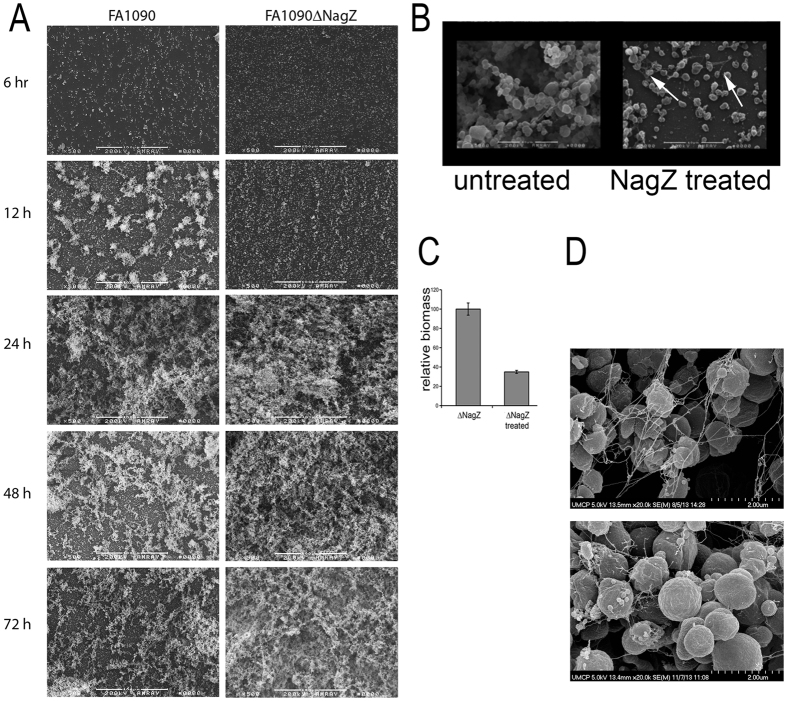
Effect of NagZ on gonococcal biofilms. Panel (**A**) SEM showing organization of biofilms produced over time (magnification 250X). Panel (**B**) is an SEM of FA1090∆nagZ biofilms, treated and untreated with exogenously added NagZ (Magnification 5000 X). Arrows in the panel indicate that NagZ treatment did not remove DNA strands. Panel (**C**) Quantification of biofilm formed by untreated mutant strain, and mutant strain biofilm treated with purified NagZ, using crystal violet method. There is a statistically significant reduction in biofilm on treatment with NagZ (p < 0.001). Two-tailed t-test assuming unequal variance was employed to determine statistical significance. Panel (**D**) shows the presence of extracellular DNA in both strains’ biofilms (magnification 20000X). The top panel is FA1090 and the lower panel is FA1090∆nagZ.

We analyzed biofilm evolution over time using scanning electron microscopy. After 12 hrs, the biofilms produced by FA1090 and FA1090ΔnagZ looked different. The biofilm produced by FA1090 appeared patchy with numerous nucleation sites while the biofilm produced by FA1090ΔnagZ appeared diffuse across the entire field of view with no obvious nucleation sites (Fig. 3A). These biofilms are dramatically different in their appearance at 12 hrs. FA1090ΔnagZ biofilms showed an increase in cell density over time, while the FA1090 biofilm density seemed to peak at 48 hrs. This difference cannot be explained by differences in the growth rate as both strains give the same growth profile (see Supplemental Fig. 3A) or due to the presence of other extracellular components as the overall biomass of the mutant biofilm is greater.

We hypothesized that the differences seen in the biofilms made by FA1090 and FA1090∆nagZ were due to the localized presence of NagZ in the extracellular milieu. For this to happen, we hypothesized that NagZ could gain access to the extracellular milieu through autolysis. We analyzed the impact of exogenously added NagZ on biofilms produced by FA1090∆nagZ. Static biofilms were formed and after treatment with NagZ protein, the biofilms were visualized using SEM. NagZ treatment reduced the thickness and density of the biofilm formed by FA1090∆nagZ (Fig. 3B). The reduction in biomass was quantified, and the data indicate more than 50% of the biofilm was removed with the addition of exogenously added NagZ (Fig. 3C). Addition of NagZ to the bacterial suspension at the beginning of biofilm formation also resulted in a similar reduction in biofilm formation after 24 hr (data not shown). After NagZ treatment, strands of nucleic acid were still apparent in the biofilm (Fig. 3B); treatment with DNase not only removed the extracellular DNA but also degraded the biofilm (data not shown). The data in Fig. 3D show the presence of extracellular DNA in biofilms produced by both strains.

### The lack of *nagZ* does not alter blebbing

*N. gonorrhoeae* generates outer membrane vesicles^32^ and alterations in vesicle formation have been observed in bacteria with defects in peptidoglycan metabolism^33^. Since NagZ is involved in peptidoglycan recycling, it is possible that NagZ could affect biofilm formation indirectly by increasing the release of outer membrane vesicles. Using high resolution SEM to visualize the biofilm architecture and the ultrastructure of bacteria within the biofilm, we determined the number and size of Blebs made by FA1090 and FA1090∆nagZ. We found that there was no difference in bleb size or distribution between the two strains, ruling out a membrane blebbing defect as the cause for difference in biofilm formation (See supplemental Fig. 4).

### Viability of cells within a biofilm

**Figure 4:**
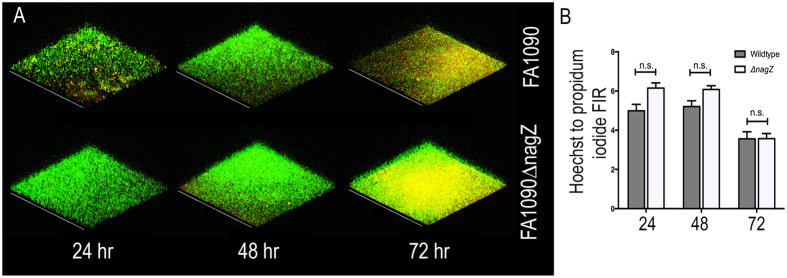
Viability of cells in gonococcal biofilms. (**A**) Static biofilms were formed with FA1090 and FA1090∆nagZ strains for different periods of time, and visualized by confocal microscope after staining with propidium iodide (red) and Hoechst (green). (**B**) The fluorescence intensity ratio (FIR) between Hoechst and PI was measured for both strains at the three time points. No significant differences were observed between the ration of dead and live cells at the various time points.

SEM images showed what appears to be DNA entangling the bacterial aggregates in a random fashion. Since FA1090 lacks the gonococcal genetic island implicated in DNA secretion^34^, the source of DNA could be through bacterial lysis or leakage of DNA from dead cells. The data in Fig. 4A show the double-stained (Hoechst and propidium iodide (PI)) biofilms visualized by confocal microscopy (Hoechst stains both dead and live bacteria whereas PI only permeates the membranes of dead bacteria). Figure 4B shows the quantification of these images by comparing the mean fluorescence intensity ratio (FIR) of Hoechst staining to PI staining. No significant difference in the proportion of live to dead bacteria was observed, although the amount of dead bacteria increased over time as seen by an increase of fluorescence intensity of PI. By 72 hrs, both biofilms contain a significant portion of dead cells.

### Effect of NagZ expression on Biofilm formation on human cells

**Figure 5:**
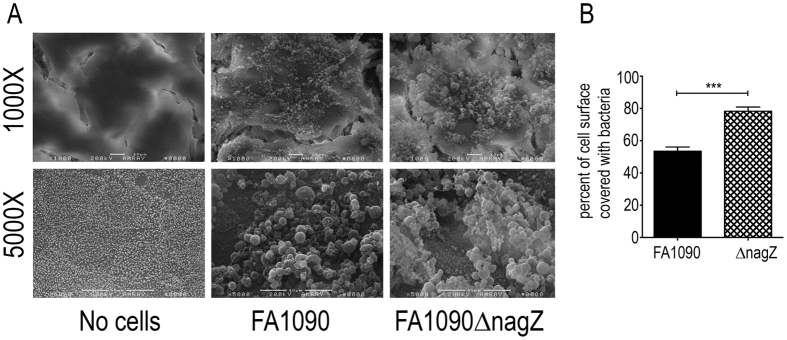
Gonococcal biofilm formation of polarized T84 cells. The left side of the figure are representative SEMs of T84 cells at different magnifications, colonized with or without added gonococci. The no cells control is untreated T84 epithelial cells showing normal morphology of these cells as visualized by SEM. The graph on the right is a quantification of the biofilms. The percent of the surface covered by biofilm was measured using ImageJ analysis. Multiple images (n = 9) were evaluated using SEM and the significance of the differences determined with Student’s t-test. (***p < 0.001).

We determined the ability of FA1090 and FA1090∆nagZ to form a biofilm on polarized T84 cells. Biofilms formed on the apical surface were visualized by SEM (Fig. 5A). The surface area covered by the biofilms was quantified using ImageJ and the percent of the area covered by biofilm determined. The data (Fig. 5B) show that the mutant strain covered significantly more surface area than the wild type strain, consistent with experiments performed on glass coverslips. Although we could not quantify the total biomass of these two biofilms, it appeared that the FA1090∆nagZ biofilm was taller than the FA1090 biofilm.

**Figure 6:**
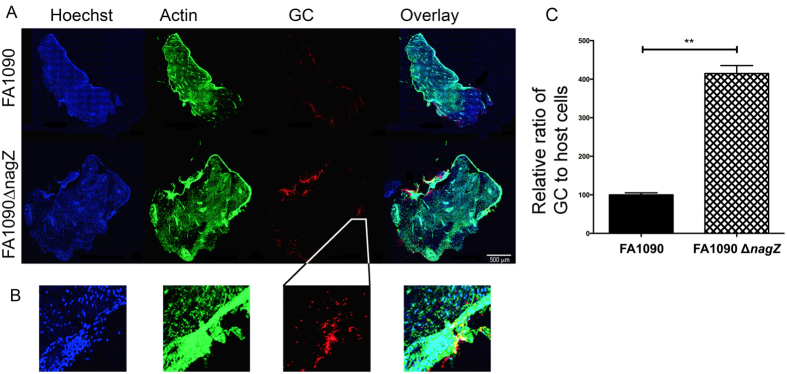
Gonococcal biofilm formation on cervical explants. Gonococci were allowed to form biofilms for 48 hrs on cervical explant tissue and after fixation visualized by confocal microscopy. Panel (**A**) shows the tissue stained for DNA (Hoechst - blue), actin (phalloidin - green), and gonococci (anti-GC antibody - red). The right most part of panel (**A**) shows an overlay of all channels. Scale bar corresponds to 500 μm. Panel (**B**) is an enlargement of the same area as shown in panel (**A**) to allow for visualization of distinct nuclei. All enlarged areas are derived from the same position as indicated for GC. The biomass of GC on cervical explant tissue was measured by normalizing the ratio of mean fluorescence intensity (MFI) of gonococci on the biofilm to the relative nuclear staining. Samples from the wild type strain were normalized to 100%. Results are average of 10 independent fields from two independent experiments. The significance of the differences was determined with Student’s t-test. (***p < 0.001).

To establish the clinical relevance of NagZ in biofilm formation, we obtained cervical tissues from women undergoing uterine surgeries and sourced by the National Disease Research Interchange (NDRI). Tissue samples were infected with ~10^9^ bacteria per tissue sample, and after 48 hr incubation, the presence of GC on the sample visualized by confocal microscopy. The Map tiling function was used to take images of small sections of the tissue as a Z-stack, and all the images were tiled using automated software to create a Z-stack of the entire cervix specimen. The stacks were Z-projected as shown in Fig. 6A. The relative number of cervical cells in a given sample area was defined based on the fluorescence intensity of the Hoescht stain (blue), which stains nuclei. The overall shape of the explant was shown by actin staining (green). The relative number of GC of in a sampled area was determined based on the red fluorescence, which was the result of immunostaining GC. The biofilm quantity was defined as the ratio of red fluorescence intensity to blue fluorescence intensity. This was then normalized to 100 (Fig. 6C). This data indicates that the *nagZ* strain produced a biofilm with four times the number of GC compared to wildtype strain, which is in agreement with experiments performed on abiotic surfaces.

## Discussion

While *N. gonorrhoeae* can form biofilms *in vivo* and *in vitro*^2^, the mechanism by which they are formed and the processes that mediate their dispersal remain unclear. We show that when GC lack the ability to make NagZ, they produce more robust biofilms that continue to accumulate over time (Fig. 2) and that exogenously added NagZ reduces these biofilms (Fig. 3). Because NagZ plays a role in peptidoglycan (PG) turnover in other organisms^23^, and is able to act in an analogous way on gonococcal peptidoglycan fragments, NagZ can be considered another example of a moonlighting enzyme^35^.

Electron microscopic studies of cervical biopsy specimens from patients with culture-proven *N. gonorrhoeae* infection have revealed evidence of biofilms; these biofilms appeared to be only a few bacteria thick^36^. It is unclear why these biofilms do not take on the qualities of those seen on abiotic surfaces or on tissue culture cells. Because FA1090∆nagZ biofilms are significantly thicker on tissue culture cells and cervical explants than FA1090, we suggest that the release of NagZ during autolysis is limiting the accumulation of biomass.

It has been hypothesized that GC biofilms produced during colonization of the cevix results in asymptomatic carriage^36^. How *N. gonorrhoeae* suppresses development of an adaptive immune response during natural infection in the presence immunostimulatory peptidoglycan fragments is unclear^37^. We suggest that removal of N-acetylglucosamine from toxic peptidoglycan monomers^38^ renders them immunosilent, and that the released N-acetylglucosamine suppresses neutrophil functions^39^ and proinflammatory cytokine induction^40^. Hence, the liberation of N-acetylglucosamine from peptidoglycan fragments by NagZ can serve to suppress the immunological signaling when the gonococcus colonizes cervical mucosa.

## Experimental Procedures

### Bacterial strains and plasmids

*N. gonorrhoeae* were grown in standard gonococcal medium (Difco, MI), designated GCP if used as phosphate-buffered broth, and designated GCK if used with agar, supplemented with 1% Kellogg’s supplement^41^, at 37 °C and 5% CO_2_. Strain F62∆8-1 has been previously described and is a strain that is genetically deleted for *lgtA*^42^. All GC strains were phenotypically non-piliated and Opa negative. *E. coli* BL21(DE3) and *E. coli* DH5αMCR (Life Technologies, MD) were grown in L broth (LB) at 37 °C or 30 °C^43^. Antibiotics included in media were used at the following final concentrations (μg ml^−1^): ampicillin 100, kanamycin 50. Plasmid pUC19 (New England Biolabs) and pET28a(+) (Novagen) were used to make deletions in various *N. gonorrhoeae* strains and to express NagZ protein, respectively. *S. aureus* SH1000 strain was obtained from Dr. Jeffery Kaplan, George Washington University.

### Chemicals and Reagents

All reagents were purchased from Sigma-Aldrich, unless stated otherwise. Polymerase chain reactions were performed using Pfu polymerase (Thermo Scientific Molecular Biology, Pittsburgh, PA) or GoTaq polymerase (Promega, Madison, WI), and carried out according to the manufacturer’s instructions. All restriction enzymes were sourced from New England Biolabs (Beverly, MA). A sample of Dispersin B was obtained from Dr. Jeffery Kaplan, George Washington University.

### Construction of mutants

A list of all primers used is provided in Table 1. A DNA fragment carrying the *nagZ* gene of *N. gonorrhoeae* FA1090 along with ~850 bp of right and left flanking DNA sequences was amplified using primers hexoF and hexoR. The resulting amplicon (~2700 bp) was cloned into the pUC19 EcoRI and HindIII sites and transformed into *E. coli* DH5αMCR, resulting in the formation of pUC19::nagZ. One of the isolated clones was used for further experiments. To disrupt *nagZ* in the gonococcus, a Kanamycin (Kan) resistance gene was excised from pK18^44^ with SmaI, the DNA fragment purified from agarose gel and inserted into the EcoRV site of pUC19::nagZ. A plasmid from a presumptive *E. coli* clone carrying pUC19::nagZ::Kan was isolated and the construct was verified by DNA sequencing (Macrogen, USA). This plasmid DNA was cleaved with EcoRV and ligated with double-strand oligo DNA prepared from DUSF and DUSR, which contain two EcoRV sites and *N. gonorrhoeae* DNA uptake sequence, after cleaving with EcoRV. Kanamycin resistant transformants were selected, the plasmid DNA isolated and checked for the presence of the uptake sequence. Plasmid DNA from such clones was used for transformation of *N. gonorrhoeae* strains by liquid transformation method^45^ and selected on GCK plates containing kanamycin (50 ug/ml). *nagZ* was deleted by a spot transformation technique^46^ in *N. gonorrhoeae* FA1090. The *nagZ* complemented strain was constructed using a PCR amplicon generated with primers hexoF and hexoR to transform the deleted strain, using a spot transformation technique^43^.

### Expression and purification of NagZ protein

DNA fragment encoding *nagZ* isolated from *N. gonorrhoeae* FA1090 was amplified using primers Ngo0135F and Ngo0135R and the resulting amplicon (1300 bp) was cloned into pET28a(+) using NheI and XhoI, resulting in the formation of plasmids pET28a(+)::nagZ. To purify NagZ, a single colony generated by transformation of *E*. *coli* BL21(DE3) with pET28a(+)::nagZ was used to inoculate 100 ml of LB broth with kanamycin. Cultures were incubated at 37 ^o^C, and when the OD_600_ of the culture reached 0.6, IPTG was added to a final concentration of 1 mM. Incubation was continued at 30 °C for an additional 12 hr. Cultures were centrifuged and bacterial pellets were resuspended in 10 ml buffer A containing 50 mM NaH_2_PO_4_ (pH 8.0), 300 mM NaCl, 20 mM imidazole, 10 mM -mercaptoethanol, 0.1% (v/v) Tween 20, 100 μM PMSF. After sonication, the cellular debris was removed by centrifugation at 40 000 *g* for 1 hr and then the supernatant applied to a 3 ml Ni-NTA agarose column previously equilibrated with 100 ml of above buffer. The column was washed with 100 ml of buffer B containing 50 mM NaH_2_PO_4_, 300 mM NaCl, 20 mM imidazole and 10% (v/v) glycerol and the proteins eluted with a 6 ml of buffer B containing increased concentration of imidazole (20 mM, 50 mM, 100 mM, 150 mM, 200 mM and 250 mM). N-acetyl-β-D-hexaminidase eluted at 150 mM–200 mM. The fractions containing the purified proteins were pooled and dialyzed against buffer B and reapplied to a Ni-NTA agarose column. The purity of the enzyme was determined by running an aliquot on a 15% SDS-PAGE. PageRuler^TM^ prestained protein ladder (170, 130, 100, 70, 55, 40, 35, 25, 15 and 10 kDa) (Fermentas) was used as protein molecular weight marker.

### LOS purification and analysis

LOS was purified from broth-grown cells by hot phenol-water method^47^. LOS was then concentrated by lyophilization and extraction with hot phenol-water continued until the preparation obtained minimal absorbance at 200 nm. LOS was analyzed on a 16.5% Tris-Tricine gel at a fixed current of 0.03 mA in an ice-cooled chamber, and visualized by silver staining^48^.

### Hexosaminidase assay

Enzyme activity was assayed using various p-nitrooligosaccharides as substrate. The standard reaction mixture consisted of 20 μl of substrate solution at a final concentration of 1.2 mM, 20 μl of 1 M KPO_4_ buffer pH 8.0 (final concentration of 200 mM), 50 μl of water and 2 μg of enzyme (final concentration of 380 nM). After 30 min incubation at 37 °C, the reaction was stopped by addition of 50 μl of 200 mM glycine NaOH buffer pH 10. Water (860 μl) was then added and the release of p-nitrophenol from the substrate was monitored at 405 nm. One unit (U) of enzyme was defined as the amount of enzyme that released 1 μmol of p-nitrophenol from pNP-GlcNAc per min at 37 ^o^C.

### Biofilm formation

To prepare biofilms, bacteria from overnight cultures grown on GCK agar plates were suspended in GCP with sodium bicarbonate and Kellogg’s supplement to a final concentration of 10^8^ bacteria per mL. Dynamic biofilms were grown in new heat-sterilized glass tubes by adding 1 mL of the bacterial suspension per tube, and incubating in a rotary shaker for 48 hr. Static biofilms were obtained by making a suspension of bacteria to a Klett of 100 in GCP and adding an aliquot to culture flasks using published methods^49^. The biofilms were quantified by staining with 1% w/v crystal violet. The crystal violet stained biofilm was eluted out by dissolving in 30% acetic acid and the absorbance read at 590 nm in a spectrophotometer.

The same protocol was used to study biofilm formation on polarized epithelial cells. T84 epithelial cells were seeded at 10^5^ per transwell insert in 24 well plates and grown for 10 days. The cells were considered polarized once the transepithelial electrical resistance (TEER) levels reach above 1500 Ω.cm^2^. Bacteria was added on the apical surface and biofilm formed for different time points. For scanning electron microscopy, the transwell membrane was fixed in glutaraldehyde and the membrane cut and removed from the insert before staining.

### Confocal Microscopy analysis

Static biofilms were grown in 35 mm glass bottom microwell dish with No. 15 coverglass (MatTek, MA) for 48 hr. Biofilms were washed with PBS three times and stained with Hoechst stain for 15 min fixed with 4% PFA for 20 min visualized with Leica TCS SP5 X confocal microscope (Leica Microsystems, IL, USA).

### Scanning electron microscopy

Static biofilms were grown for 36 hr over a glass coverslip placed inside a 24-well cell culture plate. Briefly, the coverslips were gently washed with PBS, and fixed with 2% glutaraldehyde for 60 min at RT and then overnight at 4 ^o^C. The coverslips were fixed the next day using 1% osmium tetroxide, dehydrated by a series of washes with increasing concentrations of ethanol, dried by critical point drying method, and finally coated with gold-palladium alloy. Samples were visualized with Amray 1820D microscope (20 kV) and Hitachi S4700 microscope (5 kV).

### Cervical infections

Women undergoing uterine surgeries provided informed consent for the use of cervical tissue samples, which were obtained from NDRI (National Disease Research Interchange). The University of Maryland Institutional Review Board approved of the experiments and methods, which were carried out in accordance with the approved guidelines. This endocervical tissue was received within 24 h post-surgery. Samples were cut into ~2.5 cm (L) × 0.6 cm (W) × 0.3 cm (H) pieces, incubated in CMRL-1066 (GIBCO) plus antibiotics for 24 h, and switched to antibiotic-free media for 24 h. Figure 6 the tissue was infected with approximately 10^9^ bacteria per tissue sample. After a 48 hr incubation period, the tissues he tissue was fixed with paraformaldehyde, cryopreserved in gelatin, cryosectioned, and immunostained. Antibody used to label GC was as previously described^50^. Images were acquired by confocal microscopy using a map tiling function was used to take images of small sections of the tissue as a Z-stack, and all the images were tiled using automated software to create a Z-stack of the entire sample.

## Additional Information

**How to cite this article**: Bhoopalan, S. V. *et al.* *nagZ* Triggers Gonococcal Biofilm Disassembly. *Sci. Rep.* **6**, 22372; doi: 10.1038/srep22372 (2016).

## Supplementary Material

Supplementary Figures

## Figures and Tables

**Table 1:** List of Primers.

Primer Name	DNA sequence
hexoF	GCGAATTCACAGCCGCATCTCGATAC
hexoR	GCAAGCTTTTCCGCCATGATCTACAC
DUSF	ATGCGATATCGCCGTCTGAAGAATTCGATATCATCGAT
DUSR	ATCGATGATATCGAATTCTTCAGACGGCGATATCGCAT
Ngo0135F	CTAGCTAGCATGACCGTCCCCCATATTCC
Ngo0135R	CCGCTCGAGTTAAAAGGCCTCTCCGACTTTTA

## References

[b1] CDC 2013, posting date. STD Facts (http://www.cdc.gov/std/gonorrhea/STDFact-gonorrhea-detailed.htm) Accessed Jan 5, 2016. [Online].

[b2] Greiner L. L. *et al.* Biofilm Formation by *Neisseria gonorrhoeae*. Infect Immun 73, 1964–1970 (2005). 15784536 10.1128/IAI.73.4.1964-1970.2005 PMC1087446

[b3] Lewis D. A. *et al.* Phenotypic and genetic characterization of the first two cases of extended-spectrum-cephalosporin-resistant *Neisseria gonorrhoeae* infection in South Africa and association with cefixime treatment failure. J Antimicrob Chemother 68, 1267–1270 (2013). 23416957 10.1093/jac/dkt034

[b4] Ohnishi M. *et al.* Is *Neisseria gonorrhoeae* initiating a future era of untreatable gonorrhea?: detailed characterization of the first strain with high-level resistance to ceftriaxone. Antimicrob Agents Chemother 55, 3538–3545 (2011). 21576437 10.1128/AAC.00325-11 PMC3122416

[b5] Zweig M. *et al.* Secreted single-stranded DNA is involved in the initial phase of biofilm formation by *Neisseria gonorrhoeae*. Env Microbiol 16, 1040–1052 (2013). 24119133 10.1111/1462-2920.12291

[b6] Hamilton H. L., Domínguez N. M., Schwartz K. J., Hackett K. T. & Dillard J. P. *Neisseria gonorrhoeae* secretes chromosomal DNA via a novel type IV secretion system. Mol Microbiol 55, 1704–1721 (2005). 15752195 10.1111/j.1365-2958.2005.04521.x

[b7] Steichen C. T., Cho C., Shao J. Q. & Apicella M. A. The *Neisseria gonorrhoeae* biofilm matrix contains DNA, and an endogenous nuclease controls its incorporation. Infect Immun 79, 1504–1511 (2011). 21300774 10.1128/IAI.01162-10 PMC3067525

[b8] Goytia M., Dhulipala V. L. & Shafer W. M. Spermine impairs biofilm formation by *Neisseria gonorrhoeae*. FEMS Microbiol Lett 343, 64–69 (2013). 23506248 10.1111/1574-6968.12130 PMC3651792

[b9] Goytia M. & Shafer W. M. Polyamines can increase resistance of *Neisseria gonorrhoeae* to mediators of the innate human host defense. Infect Immun 78, 3187–3195 (2010). 20439477 10.1128/IAI.01301-09 PMC2897401

[b10] Ymele-Leki P. & Ross J. M. Erosion from *Staphylococcus aureus* biofilms grown under physiologically relevant fluid shear forces yields bacterial cells with reduced avidity to collagen. Appl Environ Microbiol 73, 1834–1841 (2007). 17277217 10.1128/AEM.01319-06 PMC1828840

[b11] Stoodley P. *et al.* Growth and detachment of cell clusters from mature mixed-species biofilms. Appl Environ Microbiol 67, 5608–5613 (2001). 11722913 10.1128/AEM.67.12.5608-5613.2001 PMC93350

[b12] Izano E. A., Amarante M. A., Kher W. B. & Kaplan J. B. Differential roles of poly-N-acetylglucosamine surface polysaccharide and extracellular DNA in *Staphylococcus aureus* and *Staphylococcus epidermidis* biofilms. Appl Environ Microbiol 74, 470–476 (2008). 18039822 10.1128/AEM.02073-07 PMC2223269

[b13] Mann E. E. *et al.* Modulation of eDNA release and degradation affects *Staphylococcus aureus* biofilm maturation. PLoS One 4, 0005822 (2009). 10.1371/journal.pone.0005822 PMC2688759 19513119

[b14] LeVan A. *et al.* Construction and characterization of a derivative of *Neisseria gonorrhoeae* strain MS11 devoid of all *opa* genes. J Bacteriol 194, 6468–6478 (2012). 23002223 10.1128/JB.00969-12 PMC3497525

[b15] Blake M. S., Blake C. M., Apicella M. A. & Mandrell R. E. Gonococcal opacity: lectin-like interactions between Opa proteins and lipooligosaccharide. Infect Immun 63, 1434–1439 (1995). 7890406 10.1128/iai.63.4.1434-1439.1995 PMC173171

[b16] Cantarel B. L. *et al.* The Carbohydrate-Active EnZymes database (CAZy): an expert resource for Glycogenomics. Nucl Acids Res 37, D233–238 (2009). 18838391 10.1093/nar/gkn663 PMC2686590

[b17] Macdonald S. S., Blaukopf M. & Withers S. G. N-acetylglucosaminidases from CAZy family GH3 are really glycoside phosphorylases, thereby explaining their use of histidine as an acid/base catalyst in place of glutamic acid. J Biol Chem 290, 4887–4895 (2015). 25533455 10.1074/jbc.M114.621110 PMC4335228

[b18] Sheldon W. L. *et al.* Functional analysis of a group A streptococcal glycoside hydrolase Spy1600 from family 84 reveals it is a beta-N-acetylglucosaminidase and not a hyaluronidase. Biochem J 399, 241–247 (2006). 16822234 10.1042/BJ20060307 PMC1609908

[b19] Claus H., Maiden M. C., Maag R., Frosch M. & Vogel U. Many carried meningococci lack the genes required for capsule synthesis and transport. Microbiology 148, 1813–1819 (2002). 12055301 10.1099/00221287-148-6-1813

[b20] Mack D. *et al.* The intercellular adhesin involved in biofilm accumulation of *Staphylococcus epidermidis* is a linear beta-1,6-linked glucosaminoglycan: purification and structural analysis. J Bacteriol 178, 175–183 (1996). 8550413 10.1128/jb.178.1.175-183.1996 PMC177636

[b21] Chokr A. *et al.* Correlation between biofilm formation and production of polysaccharide intercellular adhesin in clinical isolates of coagulase-negative staphylococci. Int J Med Microbiol 296, 381–388 (2006). 16753338 10.1016/j.ijmm.2006.02.018

[b22] Chaignon P. *et al.* Susceptibility of staphylococcal biofilms to enzymatic treatments depends on their chemical composition. Appl Microbiol Biotechnol 75, 125–132 (2007). 17221196 10.1007/s00253-006-0790-y

[b23] Votsch W. & Templin M. F. Characterization of a beta -N-acetylglucosaminidase of *Escherichia coli* and elucidation of its role in muropeptide recycling and beta -lactamase induction. J Biol Chem 275, 39032–39038 (2000). 10978324 10.1074/jbc.M004797200

[b24] Petersen T. N., Brunak S., von Heijne G. & Nielsen H. SignalP 4.0: discriminating signal peptides from transmembrane regions.(ed.). (Nat Methods. 2011 Sep 29; 8(10):785–6. 10.1038/nmeth.1701. 21959131

[b25] Yu N. Y. *et al.* PSORTb 3.0: improved protein subcellular localization prediction with refined localization subcategories and predictive capabilities for all prokaryotes. Bioinformatics (Oxford, England) 26, 1608–1615 (2010). 10.1093/bioinformatics/btq249 PMC2887053 20472543

[b26] Bhasin M., Garg A. & Raghava G. P. PSLpred: prediction of subcellular localization of bacterial proteins. Bioinformatics (Oxford, England) 21, 2522–2524 (2005). 10.1093/bioinformatics/bti309 15699023

[b27] Cong Q. & Grishin N. V. MESSA: MEta-Server for protein Sequence Analysis. BMC Biol 10, 1741–7007 (2012). 10.1186/1741-7007-10-82 PMC3519821 23031578

[b28] Garcia D. L. & Dillard J. P. Mutations in ampG or ampD affect peptidoglycan fragment release from *Neisseria gonorrhoeae*. J Bacteriol 190, 3799–3807 (2008). 18390650 10.1128/JB.01194-07 PMC2395056

[b29] Cheng Q., Li H., Merdek K. & Park J. T. Molecular characterization of the beta-N-acetylglucosaminidase of *Escherichia coli* and its role in cell wall recycling. J Bacteriol 182, 4836–4840 (2000). 10940025 10.1128/jb.182.17.4836-4840.2000 PMC111361

[b30] Phillips N. J. *et al.* Proteomic analysis of *Neisseria gonorrhoeae* biofilms shows shift to anaerobic respiration and changes in nutrient transport and outermembrane proteins. PLoS One 7, e38303 (2012). 22701624 10.1371/journal.pone.0038303 PMC3368942

[b31] Stepanovic S., Vukovic D., Dakic I., Savic B. & Svabic-Vlahovic M. A modified microtiter-plate test for quantification of staphylococcal biofilm formation. J Microbiol Meth 40, 175–179 (2000). 10.1016/s0167-7012(00)00122-6 10699673

[b32] Kraus S. J. & Glassman L. H. Scanning electron microscope study of *Neisseria gonorrhoeae*. Appl Microbiol 27, 584–592 (1974). 4207582 10.1128/am.27.3.584-592.1974 PMC380088

[b33] McBroom A. J., Johnson A. P., Vemulapalli S. & Kuehn M. J. Outer membrane vesicle production by *Escherichia coli* is independent of membrane instability. J Bacteriol 188, 5385–5392 (2006). 16855227 10.1128/JB.00498-06 PMC1540050

[b34] Dillard J. P. & Seifert H. S. A variable genetic island specific for *Neisseria gonorrhoeae* is involved in providing DNA for natural transformation and is found more often in disseminated infection isolates. Mol Microbiol 41, 263–277 (2001). 11454218 10.1046/j.1365-2958.2001.02520.x

[b35] Henderson B. & Martin A. Bacterial moonlighting proteins and bacterial virulence. Curr Top Microbiol Immunol 358, 155–213 (2013). 22143554 10.1007/82_2011_188

[b36] Steichen C. T., Shao J. Q., Ketterer M. R. & Apicella M. A. Gonococcal cervicitis: a role for biofilm in pathogenesis. J Infect Dis 198, 1856–1861 (2008). 18973432 10.1086/593336 PMC2682323

[b37] Mavrogiorgos N., Mekasha S., Yang Y., Kelliher M. A. & Ingalls R. R. Activation of NOD receptors by *Neisseria gonorrhoeae* modulates the innate immune response. Innate Immun 20, 377–389 (2014). 23884094 10.1177/1753425913493453 PMC3880408

[b38] Chan Y. A., Hackett K. T. & Dillard J. P. The lytic transglycosylases of *Neisseria gonorrhoeae*. Microb Drug Resist 18, 271–279 (2012). 22432703 10.1089/mdr.2012.0001 PMC3412582

[b39] Ma L. *et al.* Immunosuppressive effects of glucosamine. J Biol Chem 277, 39343–39349 (2002). 12176986 10.1074/jbc.M204924200

[b40] Largo R. *et al.* Glucosamine inhibits IL-1β-induced NFκB activation in human osteoarthritic chondrocytes. Osteoarthritis and Cartilage 11, 290–298 (2003). 12681956 10.1016/s1063-4584(03)00028-1

[b41] White L. A. & Kellogg D. S. Jr. *Neisseria gonorrhoeae* identification in direct smears by a fluorescent antibody counterstain method. Appl. Microbiol. 13, 171–174 (1965). 14325874 10.1128/am.13.2.171-174.1965 PMC1058216

[b42] Song W., Ma L., Chen W. & Stein D. C. Role of lipooligosaccharide in *opa*-independent invasion of *Neisseria gonorrhoeae* into human epithelial cells. J Exp Med 191, 949–959 (2000). 10727457 10.1084/jem.191.6.949 PMC2193109

[b43] Sambrook J. & Russell D. W. Molecular cloning. A laboratory manual (3rd ed.).(ed.). (Cold Spring Harbor Laboratory Press, 2001).

[b44] Pridmore R. D. New and versatile cloning vectors with kanamycin-resistance marker. Gene 56, 309–312 (1987). 3315864 10.1016/0378-1119(87)90149-1

[b45] Stein D. C., Petricoin I. E. F., Griffiss J. M. & Schneider H. Use of transformation to construct *Neisseria gonorrhoeae* strains with altered lipooligosaccharides. Infect. Immun. 56, 762–765 (1988). 3126141 10.1128/iai.56.4.762-765.1988 PMC259367

[b46] Gunn J. S. & Stein D. C. Use of a non-selectable transformation technique to construct a multiple restriction modification deficient mutant of *Neisseria gonorrhoeae*. Mol. Gen. Genet. 251, 509–517 (1996). 8709956 10.1007/BF02173639

[b47] Westphal O. & Jann K. Bacterial lipopolysaccharides: extraction with phenol-water and further applications of the procedure. Meth Carbohydr Chem 5, 83–91 (1965).

[b48] Tsai C. M. & Frasch C. E. A sensitive silver stain for detecting lipooligosaccharide in polyacrylamide gels. Anal Biochem 119, 115–119 (1982). 6176137 10.1016/0003-2697(82)90673-x

[b49] Yi K., Rasmussen A. W., Gudlavalleti S. K., Stephens D. S. & Stojiljkovic I. Biofilm formation by *Neisseria meningitidis*. Infect Immun 72, 6132–6138 (2004). 15385518 10.1128/IAI.72.10.6132-6138.2004 PMC517562

[b50] Bish S. E., Song W. & Stein D. C. Quantification of bacterial internalization by host cells using a b-lactamase reporter strain: *Neisseria gonorrhoeae* invasion into cervical epithelial cells requires bacterial viability. Microbes Infect 10, 1182–1191 (2008). 18678271 10.1016/j.micinf.2008.06.014 PMC2617741

[b51] Yamasaki R., Bacon B. E., Nasholds W., Schneider H. & Griffiss J. M. Structural determination of oligosaccharides derived from lipooligosaccharide of *Neisseria gonorrhoeae* F62 by chemical, enzymatic, and two-dimensional NMR methods. Biochem. 30, 10566–10575 (1991). 1931980 10.1021/bi00107a028

