# Supplementary material for: nagZ Triggers Gonococcal Biofilm Disassembly
Source: Sci Rep. 2016 Mar 1;6:22372. doi: 10.1038/srep22372 (PMC4772129; doi:10.1038/srep22372)
Supplement: Supplementary Figures [file srep22372-s1.doc]

*nagZ* Triggers Gonococcal Biofilm Disassembly

Senthil V. Bhoopalan^1^, Andrzej Piekarowicz^1^, Jonathan D. Lenz^2^, Joseph P. Dillard^2^, and Daniel C. Stein^1^*

Supplemental Figures

**SFig. 1. Genomic organization of *nagZ* region.** Panel A represents the genomic organization of the *nagZ* region of the pathogenic *Neisseria* and nucleotide alignment of *nagZ* from all sequenced gonococci (NC_002946.2, NC_011035.1, ABZF01000028.1, ABZG01000030.1, ABZH01000027.1, ABZI01000026.1, ABZL01000026.1, ABZM01000035.1, ABZN01000023.1, ABZO01000044.1, ABZP01000042.1, ABZQ01000043.1, ACIG01000102.1, ADAA01000005.1, NC_017511.1, AKCG01000001.1, AKCH01000001.1, ABZJ02000001.1, and NC_022240.1.). The height of the green bars correspond to identity of the sequences for each nucleotide among the sequenced *N. gonorrhoeae.* Panel B is an amino acid alignment showing that gonococcal NagZ is homologous to NagZ proteins from *E. coli* and *P. aeruginosa*, and that it contains the conserved GH3 dyad sequence and the consensus active site sequence for GH3 type glycosidases. Gonococcal NagZ is 39% identical and 55% similar to *E. coli* NagZ, and 41% identical and 56% similar to *P. aeruginosa* NagZ. The tall green bars represent identical amino acids, and smaller yellow bars represent similar amino acids. Panel C shows the sequence comparison of the GH3 dyad sequence from *E. coli*, *P. aeruginosa* and *N. gonorrhoeae.*

**SFig. 2. Biochemical analysis of NagZ.** Panel A: SDS-PAGE gel of purified NagZ. Lane 1 is the molecular weight standard (mass of each band is indicated on the left of the gel) and lane 2 is purified NagZ. Panel B: the activity profile for NagZ as assayed over a variety of temperatures. Panel C: the activity profile for NagZ as assayed over a variety of pHs. For panels B and C, the activity of NagZ used p-Nitrophenyl N-acetyl-β(1-4)-D-glucosaminide as a substrate. Panel D: the relative activity of NagZ for various substrates measured at 37ºC at a pH 8.0. Panel E: a biofilm of *S. aureus* SH1000 was incubated with Dispersin B (10 μg) or NagZ (20 μg), and the amount of biofilm remaining in the well determined by measuring the biomass using a crystal violet staining procedure.. Data represents mean values (± SE) of three independent experiments performed in triplicate. Statistical significance was determined using two-tailed t-test assuming unequal variance. (*** p < 0.001).

**SFig. 3. NagZ is found in culture supernatants of stationary phase grown gonococci.** Panel A shows a growth curve of FA1090 and FA1090ΔnagZ, monitored hourly by measuring the turbidity using a Klett-Summerson colorimeter. Liquid broth (1 ml) was collected every 2 hours and assayed for presence of NagZ, using the methods described in the materials and methods. The relative levels of NagZ activity measured from supernatant are shown. Panel B shows NagZ activity measured using pNP-GlcNAc as a substrate in supernatants and bacterial lysates from mid-log phase wild type and mutant cultures. Activity was normalized to 100% of the activity obtained from the wild-type strain after cell lysis of a 24 hr culture. Statistics were two tailed t-test (*** p < 0.001).

**SFig. 4. Bleb formation by FA1090 and FA1090∆nagZ.** Panel A: High magnification image of gonococcal clusters found within the biofilm at different time points (20,000x). These images are representative of bacteria when they are contained within a cluster. Panel B: Average number of OMV blebs per field in biofilm formed by FA1090 and FA1090ΔnagZ. Multiple SEM images of 48 hr biofilms were acquired at 20,000x magnification. Membrane blebs were defined as anything less than 250 nm and counted using ImageJ software. (n.s. – not significant).

Supplemental figure 1


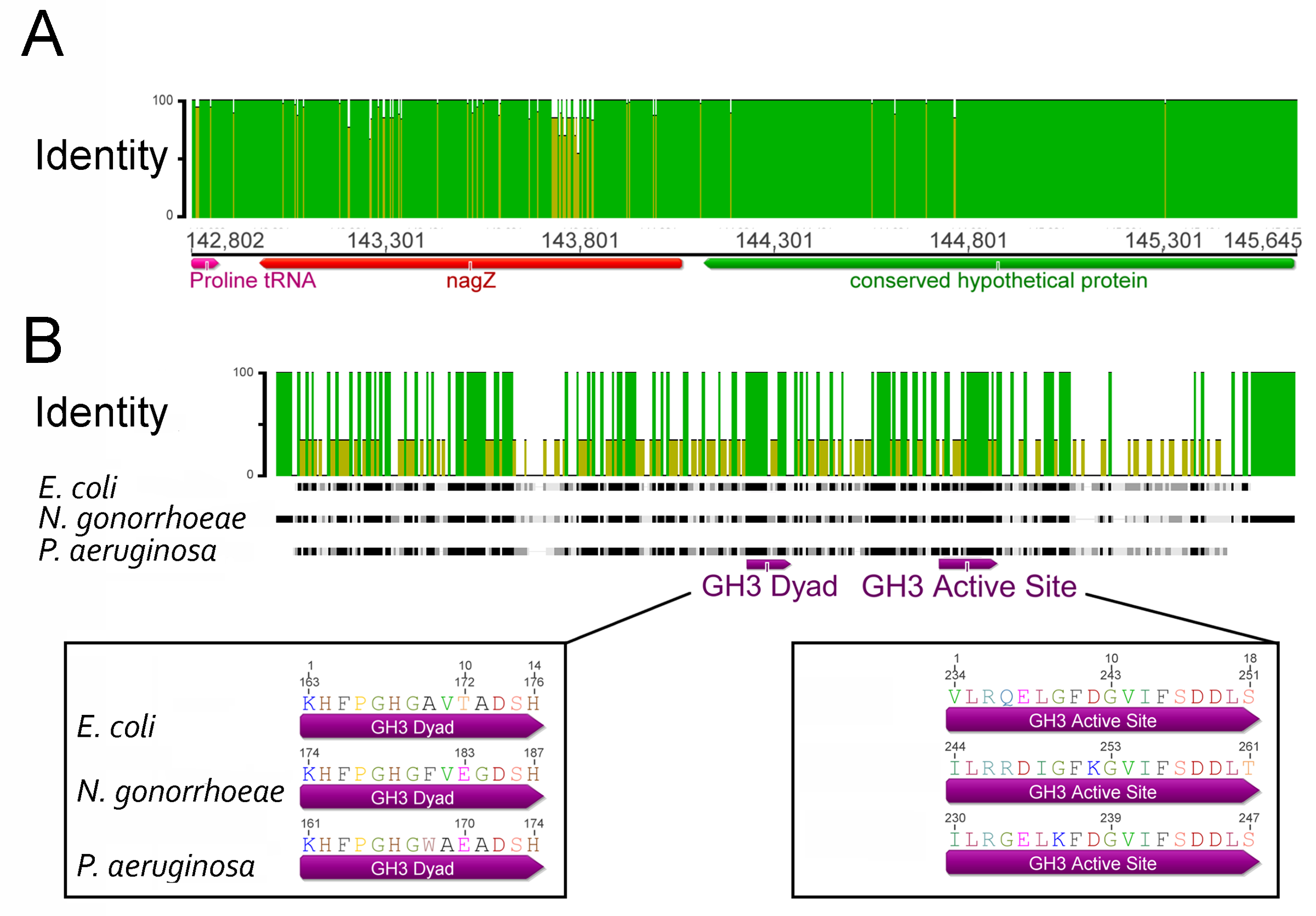


Supplemental Figure 2


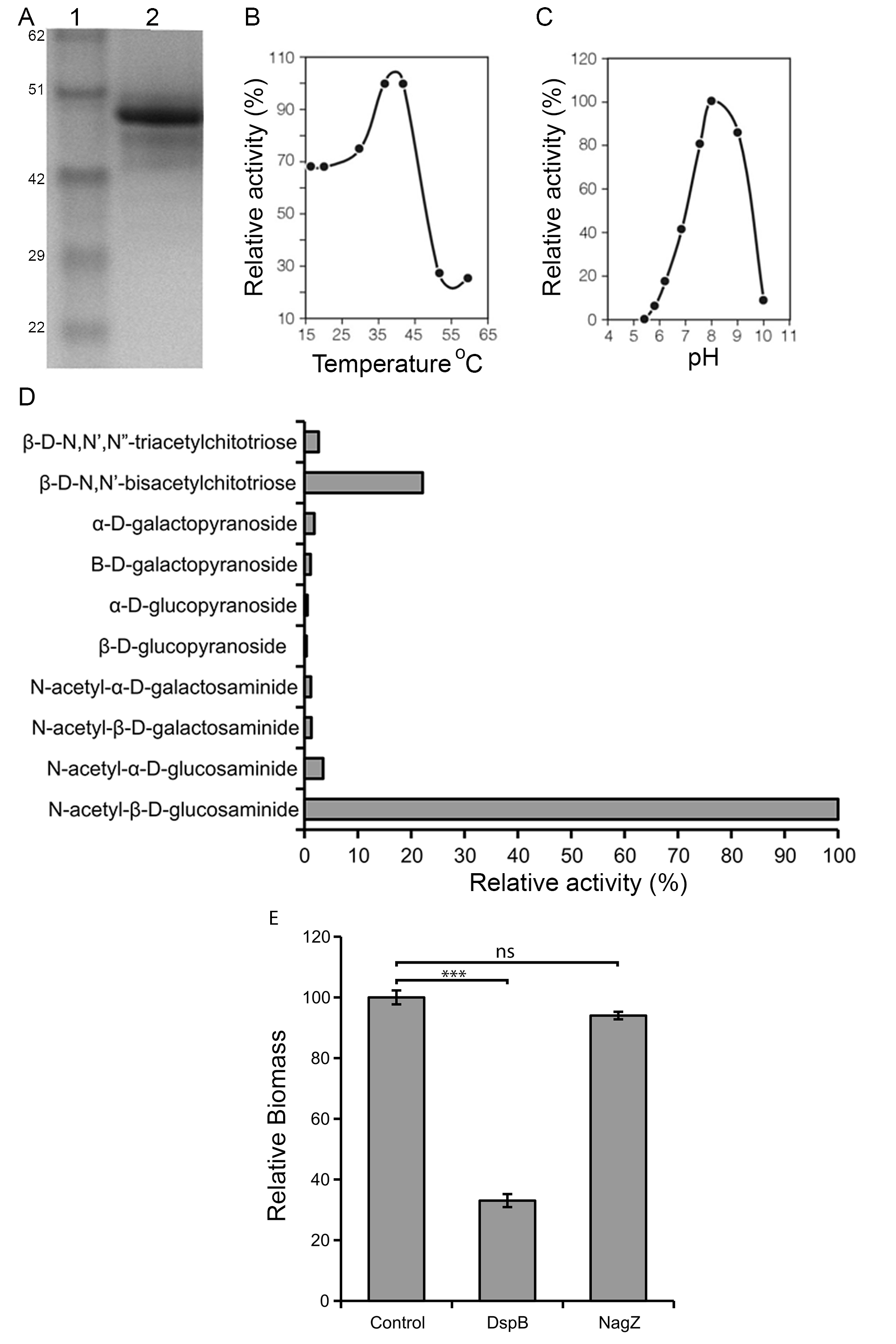


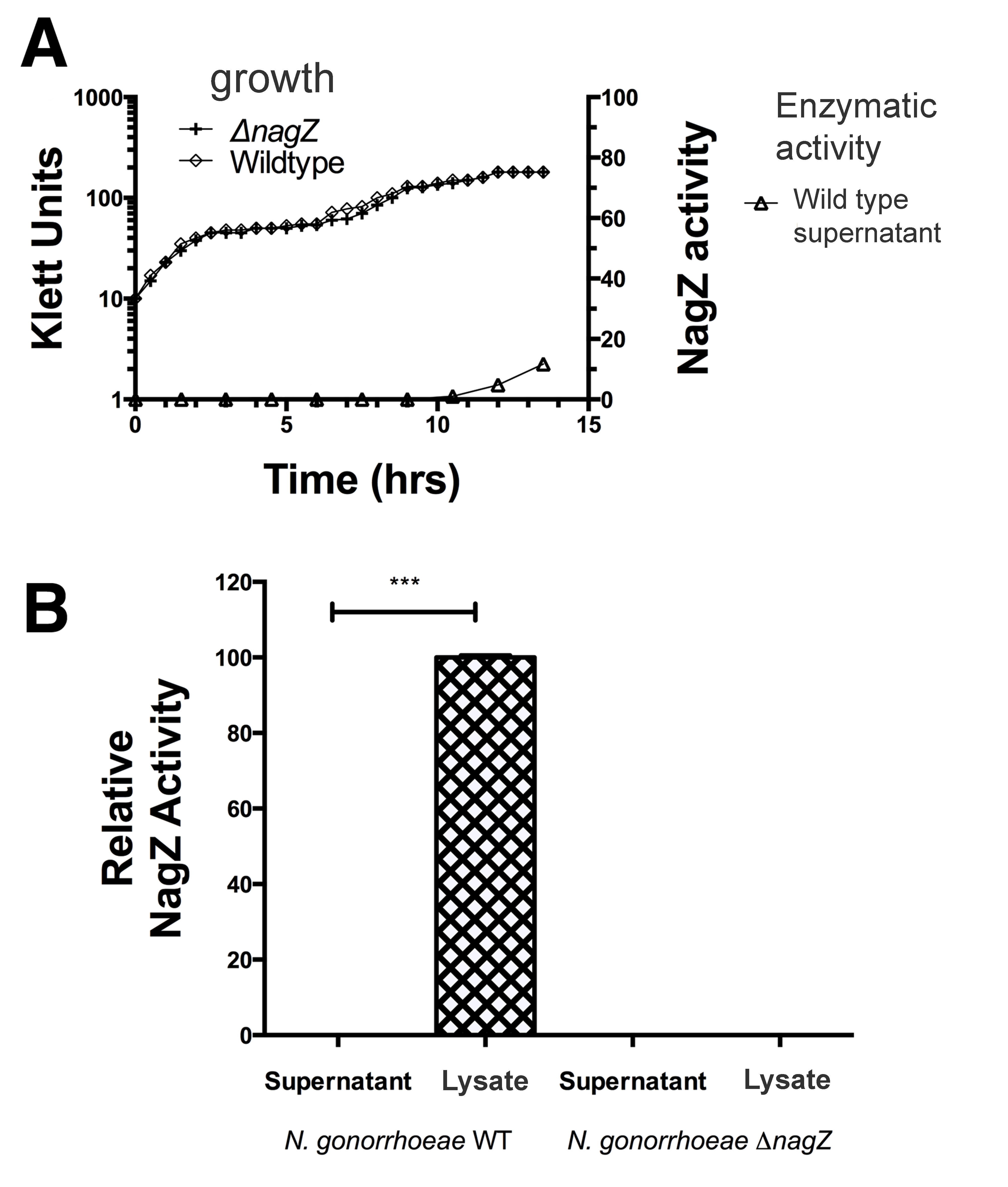
Supplemental Figure 3


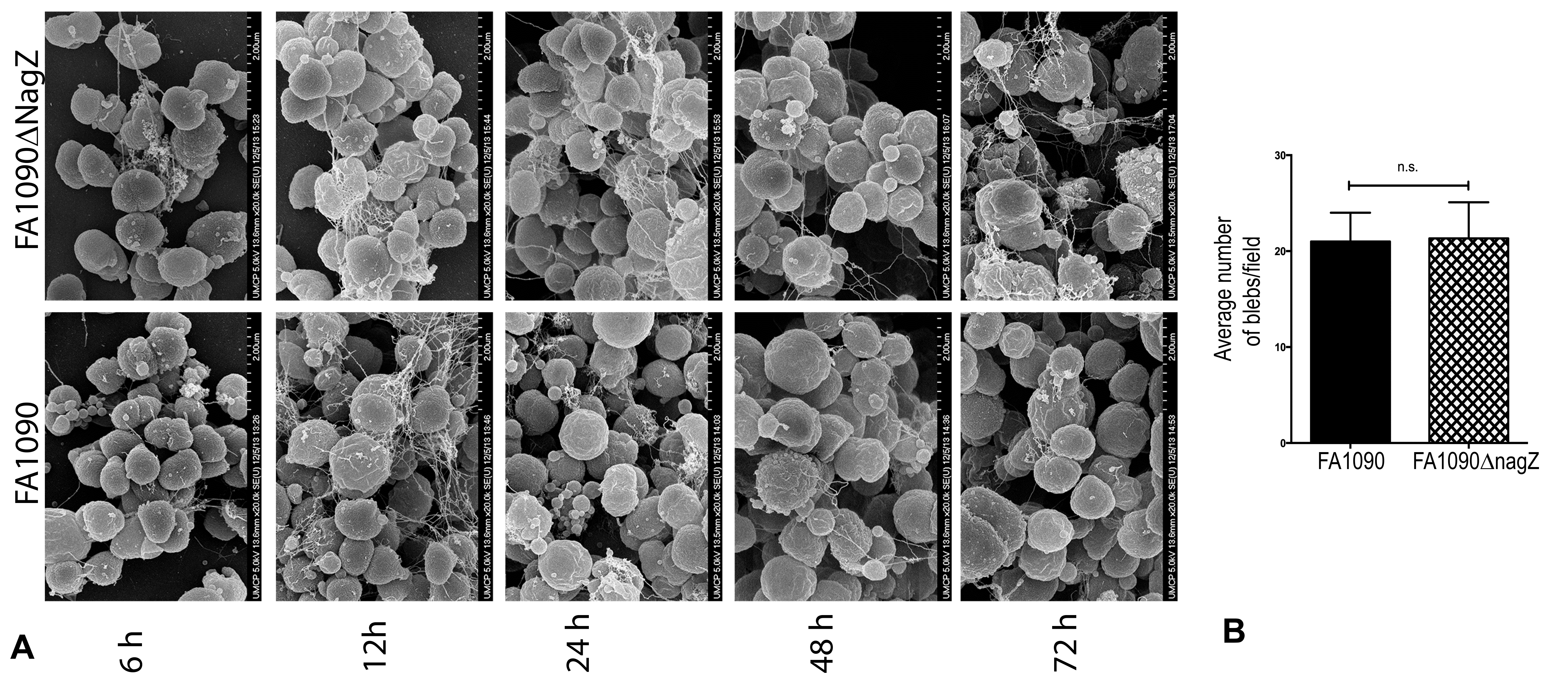
Supplemental figure 4
